# Gravity Influences Top-Down Signals in Visual Processing

**DOI:** 10.1371/journal.pone.0082371

**Published:** 2014-01-06

**Authors:** Guy Cheron, Axelle Leroy, Ernesto Palmero-Soler, Caty De Saedeleer, Ana Bengoetxea, Ana-Maria Cebolla, Manuel Vidal, Bernard Dan, Alain Berthoz, Joseph McIntyre

**Affiliations:** 1 Laboratory of Neurophysiology and Biomechanics of Movement, ULB Neuroscience Institut, Université Libre de Bruxelles, Brussels, Belgium; 2 Laboratory of Electrophysiology, Université de Mons, Mons, Belgium; 3 Laboratoire de Physiologie de la Perception et de l'Action, CNRS Collège de France, Paris, France; 4 Centre d'Etude de la Sensorimotricité (UMR 8194), Institut Neurosciences et Cognition, CNRS - Université Paris Descartes, Paris, France; University of British Columbia, Canada

## Abstract

Visual perception is not only based on incoming visual signals but also on information about a multimodal reference frame that incorporates vestibulo-proprioceptive input and motor signals. In addition, top-down modulation of visual processing has previously been demonstrated during cognitive operations including selective attention and working memory tasks. In the absence of a stable gravitational reference, the updating of salient stimuli becomes crucial for successful visuo-spatial behavior by humans in weightlessness. Here we found that visually-evoked potentials triggered by the image of a tunnel just prior to an impending 3D movement in a virtual navigation task were altered in weightlessness aboard the International Space Station, while those evoked by a classical 2D-checkerboard were not. Specifically, the analysis of event-related spectral perturbations and inter-trial phase coherency of these EEG signals recorded in the frontal and occipital areas showed that phase-locking of theta-alpha oscillations was suppressed in weightlessness, but only for the 3D tunnel image. Moreover, analysis of the phase of the coherency demonstrated the existence on Earth of a directional flux in the EEG signals from the frontal to the occipital areas mediating a top-down modulation during the presentation of the image of the 3D tunnel. In weightlessness, this fronto-occipital, top-down control was transformed into a diverging flux from the central areas toward the frontal and occipital areas. These results demonstrate that gravity-related sensory inputs modulate primary visual areas depending on the affordances of the visual scene.

## Introduction

Gravity plays a crucial role in building a neural representation of physical space [1], [2], [3]. The perception of spatial orientation, both static and during motion, depends on integration of afferent signals from the vestibular organs with visual, proprioceptive and tactile inputs [4], [5]. Moreover, electrical stimulation of the lateral temporo-parietal area induces pitch or yaw plane illusions [1], demonstrating a high level of spatial plane integration in the brain. This region participates in a network that is activated by visual motion and by vestibular stimulation. This “vestibular network” is composed of the temporo-parietal junction, the cingulate cortex, the ventral premotor area, the supplementary motor area, the middle and post-central gyrus, the posterior thalamus and the putamen [1], [6], [7]. It has been demonstrated [2] that this network is involved in processing visual motion when it is coherent with natural gravity, supporting the hypothesis that the fundamental physical constraint of Earth's gravity is internalized by the human brain [8].

Human visual recognition processes are robust; they can provide the perception of real motion even during virtual navigation [5]. Visual processing relies on an integrated, multimodal reference frame, including vestibular and proprioceptive inputs, thereby recreating complex behaviors from visual inputs alone. The emergence of a unified percept depends on the coordination of clusters of neuronal networks widely distributed in the brain [9] and is influenced by the spatial environment, experience relating to image content, sense of minimal self and state of action [10], [11]. In non-human primates, different cortical areas are known to combine multiple sensory inputs that are dynamically re-weighted to maintain behavioral goals [12] while in humans, task-related aspects represented in the prefrontal cortex modulate sensory processing by a top-down process acting on the visual cortex [13].

We hypothesized that cortical visual processing would be altered when gravitational cues are suppressed, but only when task goals imply the need for self-motion. In particular, weightlessness could significantly change early visual evoked potentials through reciprocal interactions with cerebral regions involved in multisensory integration (top-down). To test this hypothesis, we compared visual evoked potentials (VEPs) triggered by a classical checkerboard-reversal pattern (neutral stimulus) with those induced by the presentation of a view inside a 3D tunnel during a virtual navigation task, both on Earth and in weightlessness. Because it has been recently reported that top-down modulation is supported by phase coherence of electroencephalographic (EEG) signals between the prefrontal cortex involved in attentional processes and the visual areas implicated in early VEPs [13], we applied, in conjunction with the evoked response, analysis of the event related spectral perturbation (ERSP), inter-trial coherency (ITC) [14], [15] and the imaginary part of the coherency [16], [17] in two different conditions, on Earth and in the International Space Station (ISS).

## Materials and Methods

### Participants

Five male astronauts participated in this investigation. Each astronaut was tested on Earth before their spaceflight, in weightlessness aboard the ISS, and soon after the return to Earth. These experiments were performed during the joint Russian-Belgian ODISSEA and INCREMENT 9 and 10 missions. The mean age (± SD) of the astronauts was 42±3 years. All astronauts were in excellent health, as regularly determined by a special medical commission during all periods of the investigation. Following the stay in ISS, astronauts reported on eventual medication use and sleep quality aboard the ISS. In accordance to the Declaration of Helsinki, the European Space Agency Medical Care Committee approved all experimental procedures. All subjects gave written informed consent.

The specific schedule of testing was as follows. Prior to flight, astronauts were tested on Earth in 2 pairs of sessions over the 2 months preceding lift-off. In flight, astronauts were tested on two days over the course of their space flight. They were then tested again back on Earth, on at least 2 days during the week immediately following the landing and two more times one to three weeks later.

### Procedure

Participants looked straight ahead through a form-fitting facemask and a circular barrel (cylinder) at the laptop screen. The screen was centered on the line of gaze at a distance of ∼30 cm from the eyes. Viewing through the barrel removed any external visual references. A strap attached to the facemask passed behind the head to help keep the facemask firmly in place against the subject's forehead. A trackball was mounted on the right side of the barrel, such that the subject could hold onto the entire structure (mask/barrel/laptop) with both hands and still manipulate the trackball with the thumb.

On Earth, subjects performed the experiment while seated upright in front of the computer. The laptop was placed on a support table such that the facemask was at eye height when the subject was in a comfortable, upright, seated position. During space flight, they performed the experiment in two conditions. In the attached condition, the astronauts used belts, foot straps and a tabletop to reproduce a seated posture that was essentially the same as that used on Earth. In the free-floating condition, participants held the experimental apparatus between the two hands such that both participant and apparatus floated free from any contact with the station. A second astronaut served as a spotter during these tests to ensure that the subject did not drift into contact with the walls, floor or ceiling of the ISS module. If the subject did start to drift toward contact with the station, the spotter tugged lightly and briefly on the clothing of the subject so as to cancel the drift, but in a way that avoided giving any orientational cues or significant accelerations.

### Stimuli and Tasks

All participants performed two tasks: 1) passive observation of a checkerboard reversal pattern and 2) viewing of a 3D virtual tunnel and a virtual movement through the tunnel in view of reporting the perception of the bend in the tunnel.

#### Checkerboard Reversal Pattern

Checkerboards with EGA graphic resolution were sequentially presented in pattern reversal mode on the high-resolution screen of an IBM Laptop (screen of 22.0 cm height, 30.3 cm width; refresh rate of 75 Hz, resolution of 640×480 pixels). The display subtended 7°(w)×5°(h) at the eye. Thus, both foveal and parafoveal retinal fields were stimulated. Visual stimuli presented on this display consisted of black and white rectangles measuring 3.85 cm in width by 2.80 cm in height. The checkerboard contrast was 50% and the stimulation frequency was 3 Hz. Because of the severely limited amount of crew time available aboard the ISS, the duration of checkerboards test was limited to 30 s. With a fixed inter-stimulus interval of 333 ms this resulted in 88 usable reversals of the checkerboard. We collected one such sequence of stimuli during each experimental session on the ground and once during each session and postural condition (attached or free-floating). The checkerboard test was always performed just prior to performing the virtual navigation task to be described below within each session and each postural condition.

#### 3D Virtual Tunnel

The images of the 3D virtual tunnel were non-stereoscopic but included perspective cues generated by the OpenGL graphic libraries (more details are given in Vidal et al. [5]). The virtual navigation test was performed 48 times and the duration of one passage through the tunnel was about 12 s.

The sequences of a single trial in the navigation test was the following: (1) When ready, the participant initiated the trial by pressing a button to trigger the appearance of a black screen with a central green spot that he then had to fixate. (2) After one second, a static image of the entrance to the tunnel was presented for one second. This transition time-event between the black screen and the static image triggered the evoked response studied in the present study. (3) The onset of the movement of the virtual navigation occurred at the end of the 1 s static period, thus well after the period during which the EEG was analyzed. (4) Participants were ‘driven’ passively through a virtual tunnel with stone-textured walls in the form of a bent pipe with a constant-radius circular cross-section. Movement through the tunnel took 7 seconds [5]. At the end of the virtual movement through the bent pipe subjects were asked to report their perception of the bend's angular magnitude by adjusting, with a trackball, the angular bend in a rod symbolizing the outside view of the tunnel. The time to produce the response varied from subject to subject and from trial to trial, but was typically on the order of 4 seconds.

Subjects thus viewed the visual image of the entrance to the tunnel in the context of a cognitive task and were thus encouraged to maintain their level of attention throughout the movement. Here, only the static images of the initial presentation of the 3D tunnel images presented during 1 s before the onset of the virtual movement were taken into account. More precisely, we analysed the potentials evoked by the transition from the grey screen with the fixation dot to the initial, static image of the entrance to the tunnel, and we analysed the frequency content and phase of the signals around the time of the transition (see below). In this article we report only the EEG responses to the static image of the tunnel. The analysis of the perceptual responses was reported previously [18] and the EEG activity during the virtual movement will be reported elsewhere.

Given the timing of each of the steps in a single trial, and the variable time that it took the subjects to produce the cognitive response, the inter-stimulus interval for the initial image into the tunnel varied between 12 and 16 seconds, resulting in a frequency of the appearance of the static image that we studied here ranging from 0.06 Hz to 0.08 Hz.

### EEG recordings and analysis

The electroencephalogram (EEG) of the astronauts was measured using a cap equipped with electrodes (Electro-Cap adapted for the ISS, see Neurocog ESA mission) in which at least 14 Ag–AgCl electrodes were placed at positions F7, F3, Fz, F4, F8, C3, Cz, C4, T5, P3, Pz, P4, O1, O2, according to the international 10–20 system. All of the electrodes were referenced to linked mastoids. Scalp electrode impedances were measured and kept below 5 KΩ.

The EEGs were filtered with an analogue band-pass of 0.01–100 Hz and sampled at 256 Hz. Each trial contained samples from −0.1 s before to 0.4 s after the onset of stimulus for the checkerboard condition and from −0.5 s before to 1.0 s after for the 3D tunnel condition.

Blinks and eye movements (horizontal and vertical components) were monitored with electrodes at the outer canthi of the eyes (horizontal electrooculogram, EOG) and above and below the right eye (vertical EOG). Ocular artifacts were removed using EEGLAB ICA routine [19], [20]. Remaining events containing other types of artefacts were rejected by using the EEGLAB artefact rejection routines (http://sccn.ucsd.edu/wiki/Chapter_01:_Rejecting_Artefacts). After this procedure, 1 to 4% of the checkerboard trials and 23 to 31% of 3D tunnel trials (all conditions confounded) were rejected, respectively. Because evoked studies of evoked potentials require a sufficient number of trials – and after checking that there are no significant differences in electrophysiological responses between these two space flight conditions – data from the attached and free-floating conditions during space flight were pooled together.

### Visual evoked potentials

Visual evoked potentials (VEP) were measured at the occipital (O2) and frontal (F8) loci with respect to the reference electrode placed on the right earlobe. For each recording condition the peak latency and the related absolute amplitude were measured for the main VEP components P1 and N1 [23]. These peaks were extracted automatically by selecting the maximum/minimum over the [80–120] ms and the next minimum/maximum over the window [120–200] ms.

### Event-related spectral perturbation (ERSP)

The EEGLAB software [21] allows one to analyze event-related dynamics and to decipher the ongoing EEG processes that may be partially time-and phase-locked to experimental events. The event-related spectral perturbation measure (ERSP) may correspond to a narrow-band of event-related de-synchronization (ERD) or synchronization (ERS)). Briefly, for this calculation, the EEGLAB computes the power spectrum over a moving sliding latency window, and then performs averaging across data trials. A color code at each image pixel indicates the power achieved (in dB) at a given frequency and latency relative to the stimulation onset. Typically, for n trials, if 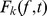 represents the spectral estimate of *k^th^* trial at frequency *f* and time *t* the ERSP can be computed as follows:
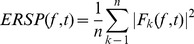
(1)To compute 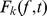, we used the short-time Fourier transform option provided in the EEGLAB software.

### Inter-trial (phase) coherence (ITC)

ITC is a time-frequency domain magnitude that indicates the degree of phase synchronization at a particular latency and frequency to a set of experimental events to which EEG data trials are time locked. This measure, also called ‘phase locking factor’ in Tallon-Baudry et al. [22], is defined as:
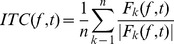
(2)where |•| represents the complex norm. The ITC measure takes values between 0 and 1. A value of 0 represents an absence of phase synchronization between EEG data and the time locking events; a value of 1 indicates perfect phase synchronization.

### Coherency

The method developed by Nolte et al. [17] allows to determine brain connectivity from quantities that are unbiased by non-interacting sources. We applied this method on the 8–10 Hz frequency band because the significant ERPS and ITC values were found in this frequency range for both visual stimulation conditions (see Results). Briefly, coherency between two EEG-channels is a measure of the linear relationship between two signals at a specific frequency and is computed as:
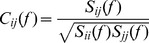
(3)where power spectra and the cross-spectrum are given by:
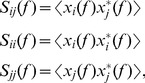
(4)and where 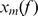 represent the Fourier transforms at frequency *f of* channel *m* for a given segment or trial, * indicates the complex conjugate of 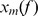 and 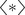
denotes the expectation value which is typically approximated by an average over the segments or trials. Then by taking the imaginary part of the coherency, 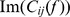, we isolate that part of coherency which necessarily reflects true interaction unbiased by non-interacting sources [17]. A coherence matrix contains an enormous amount of information; we have applied the representation developed by Nolte et al. [17] allowing a global view of all connections in one plot. In such illustrations, the large outside circle represents the whole scalp and the small single circles, also representing the scalp, containing the 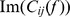 calculated for the respective electrode (indexed by a black dot) with all other electrodes.

### Directionality

In order to estimate the direction of information flux between the different EEG channels, we use the Phase Slope Index (PSI) as described in Nolte et al. [17]. This measure allows determining which channels send the information (driver) and which channels received the information (recipient). The basic idea of PSI is that interaction requires some time lag, and assuming that the speed at which different waves travel is similar, then the phase difference between sender and recipient increase with frequency and a positive slope of the phase spectrum should be expected. The characteristics that make this measure of interest in our paper are the following:

1. This quantity properly represents relative time delays of different signals and especially coincides with the classical definition for linear phase spectra.
2. It is insensitive to signals that do not interact regardless of spectral content and superposition of these signals.
3. It properly weights different frequency regions according to statistical relevance.

In mathematical term the PSI index is defined as:
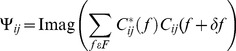
(5)where 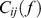 is the coherence between channel *i* and *j* given by equation (3), *δf* is the frequency resolution and Imag represents the imaginary part. *F* is the set of frequencies over which the slope is summed.

### Signal to noise ratio (SNR) and Reliability

As environmental artifacts as well as the number of averaged events [22] may decrease the SNR by increasing the noise level, we checked whether the reliability of the ERP was the same on Earth and in the ISS. The SNRs and the reliability where computed following [24], [25] as follow:
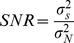
(6)where 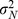 and 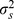 are the signal and power noise. We estimate these parameters as:
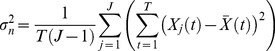
(7)and
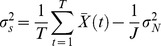
(8)where 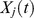 denote the EEG signals at time *t* at trial *j*, *J* and *T* denote the total number of trials and event respectively. 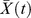 defines the average evoked potential at time *t*. Finally the reliability was computed as:
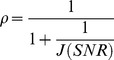
(9)


### Statistical analyses

Data from the visually evoked potential (VEP), inter-trial phase coherence (ITC) and event-related spectral perturbation (ERSP) analyses were submitted to nonparametric Friedman ANOVA to compare multiple dependant samples with *recording period* (before, during and after spaceflight) as a within-subject factor. If the test (p<0.05) results were significant, planned comparisons were made using the Wilcoxon Matched Pairs Test.

To assess whether specific topographic maps in the coherency were significant we use the non-parametric permutation method developed by Nichols and Holmes [26]. For our experiment we used a paired t-test to compare samples carried out in each order, with the null hypothesis that for each subject, the experiment would have yielded the same results if the condition were arbitrarily assigned.

### EEG experiments on control participants on Earth

In order to check the possible influence of technical details related to the presentation of the two different images in the original experiment described above, we performed a control experiment on Earth in which the opening of the 3D tunnel was presented briefly, without the subsequent virtual movement through the tunnel or the need to estimate the angle of the bend. In contrast to the navigation task performed by the astronauts, no cognitive task was required of the subjects in this control experiment. We compared the responses evoked by the appearance of these images to the presentation of the checkerboard, as before. The visual stimuli were presented to the control subjects with the same apparatus (laptop and barrel frame) as the one used by astronauts.

For both images in the control experiment, (checkerboard and 3D tunnel) an identical stimulation rate (1.0 Hz) was used, which is somewhat longer than the typical presentation frequency of a checkerboard stimulus but significantly shorter than the inter-stimulus interval for the tunnel appearance in the main experiment. The presentation of each visual item (with a presentation time of 500 ms) was immediately followed by the presentation of a neutral gray pattern (also for 500 ms). A sequence of checkerboard or 3D tunnel presentations was comprised of 100 images intermixed by 100 gray patterns. Each type of sequence was repeated 3 times, alternating between the two stimulus types and separated by one minute of rest. The duration of one recording session was 12 minutes, 5 minutes for each type of visual stimulus representing a total of 300 trials and 2 minutes of rest.

In this control experiment, EEG was recorded from 64 scalp sites using shielded electrocap. All recordings were unipolar against the right earlobe and were recalculated off-line to a linked ear lobe reference. Vertical eye movements (EOG) were recorded unipolarly against the common reference and horizontal EOG was recorded bipolarly. All electrode impedances were maintained below 5 kΩ. Scalp potentials were amplified by ANT DC-amplifiers (ANT, the Netherlands) and digitized with a rate of 2048 Hz and a resolution of 16 bits (range 11 mV). Participants were asked to avoid eye blinks and to fixate the green dot presented in the middle of the screen in order to reduce eye artefacts. Only the transitions between grey pattern to the checkerboard or the 3D image were used to trigger the evoked response. The related evoked responses were analysed in 5 control participants age-matched (age±SD years) to the 5 astronauts. All gave informed consent prior to starting the experiment and were free to stop the procedure at any time.

## Results

### The checkerboard VEP, but not the 3D tunnel VEP, was preserved in weightlessness

**Figure 1:**
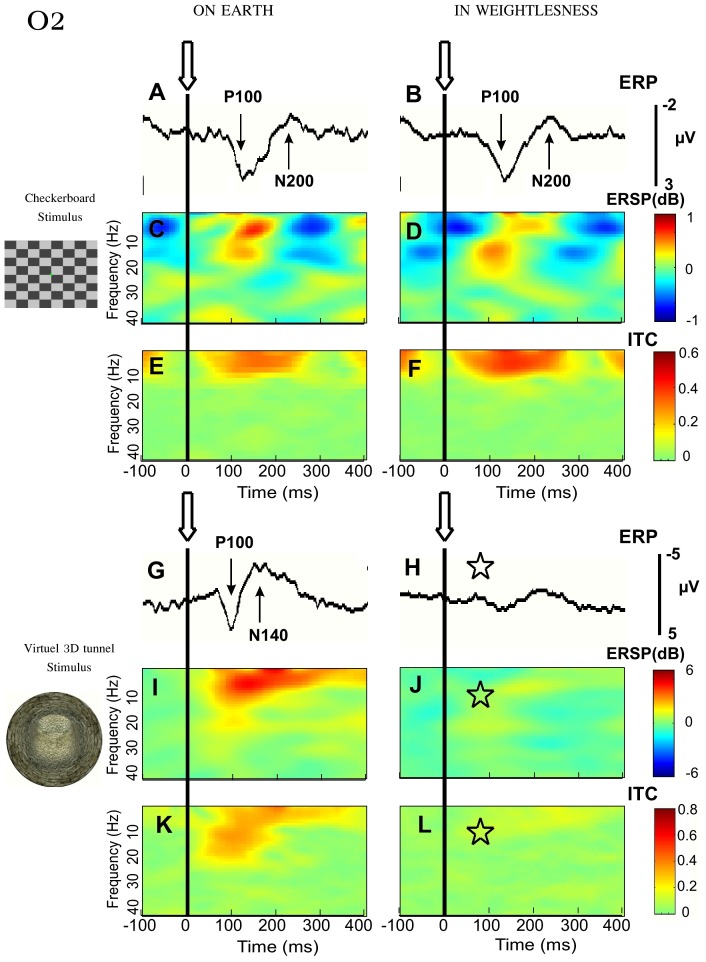
Effect of microgravity on VEP, ERSP and ITC recorded in occipital area (O2). Grand average (n = 5) triggered (arrows and vertical dashed lines) by the checkerboard-reversal pattern (***A*** *–* ***F***) and by the 3D-tunnel-image (***G*** *–* ***L***) recorded on Earth before the flight (left) and in weightlessness. Statistical significance (Friedman ANOVA) p<0.05 is indicated by an asterisk.

On Earth and in weightlessness, the 5 astronauts showed VEPs with identifiable P1-N1 components for the checkerboard-reversal (Fig. 1*A*) and for the apparition of the 3D tunnel (Fig. 1*G*) in the occipital loci (O2 channel). The latency of the peaks of P1 and of N1 differed depending on the stimulus (P1: 95±6 ms for the 3D image versus 131±25 ms for the checkerboard, p<0.0006 and N1: 145±21 ms for the 3D image versus 214±31 ms for the checkerboard, p<0.0009; using the Wilcoxon test). These differences in latency were conserved in weightlessness (p<0.0001 for P1 and p<0.00006 for N1).

The mean amplitudes of P1 and N1 measured on Earth were higher for the 3D stimulus than for the checkerboard: 7.3±3 µV versus 3.4±1.0 µV for P1 (p<0.0004) and 6.6±2.7 µV versus 2.7±1.5 µV for N1 (p<0.0001). Friedman ANOVA comparison of P1 and N1 amplitudes before, during and after spaceflight showed a significant effect of experimental conditions for the 3D tunnel but not for the checkerboard Indeed, for the checkerboard, all 5 astronauts maintained the same amplitude of the P1 and N1 components on Earth and in weightlessness, with no significant difference between the gravity conditions: 2.4±0.6 µV for P1 in weightlessness versus 3.4±1.0 µV on Earth (Chi^2^ = 4.77; df = 2; *p* = 0.09) and 1.8±0.6 µV in weightlessness for N1 versus 2.7±1.5 µV on Earth (Chi^2^ = 4.76; df = 2; *p* = 0.09) as illustrated in Fig. 1*A,B*. In contrast, the amplitude of the VEP diminished dramatically when the 3D tunnel was presented in weightlessness compared to Earth: 1.5±0.9 µV for P1 in weightlessness versus 7.3±3 µV on Earth (Chi^2^ = 19; df = 2; *p*<0.0001) and 2.1±1.0 µV for N1 in weightlessness versus 6.6±2.7 µV on Earth (Chi^2^ = 22.3; df = 2; *p*<0.0001) as seen in Fig. 1*G,H*. On return to Earth, the P1 and N1 components evoked by the 3D tunnel partially recovered, reaching an amplitude of 4.6±2.8 µV and 5.3±2 µV, respectively, which remained significantly different from the preflight values (*p* = 0.03 for P1 and *p* = 0.01 for N1, Wilcoxon test).

**Figure 2:**
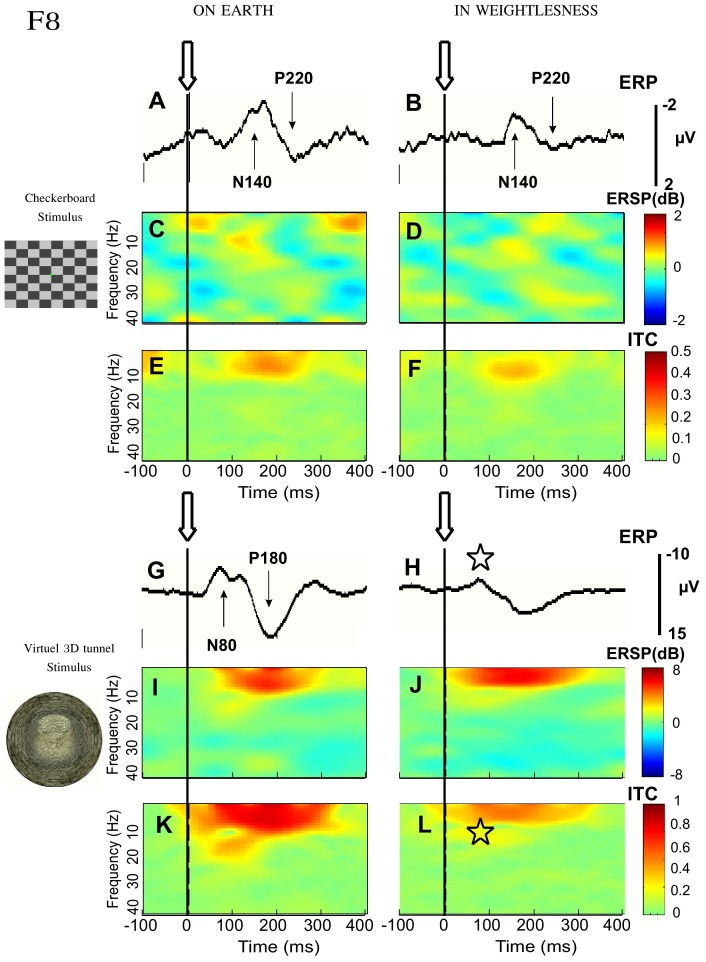
Effect of microgravity on VEP, ERSP and ITC recorded in frontal area (F8). Same disposition as in Fig. 1. Statistical significance (Friedman ANOVA) p<0.05 is indicated by an asterisk.

The same analysis of the cortical activity performed in the frontal areas (i.e. as measured by the F8 electrode) showed that as for the occipital loci, the VEP amplitudes (N1 and P1) corresponding to the checkerboard stimulation were conserved in weightlessness. There was no significant variation of either value between measurements taken before, during and after flight (Chi^2^ = 0.22; df = 2; p = 0.305 for N1 and Chi^2^ = 2.37; df = 2; p = 0.895 for P1) (Fig. 2*A–F*). In contrast, for the presentation of the 3D tunnel the N1 and P1 amplitudes did differ significantly between the measurements taken before, during and after flight (Chi^2^ = 6.12; df = 2; p = 0.04 for N1 and Chi^2^ = 18.87; df = 2; p<0.00008 for P1), with a noticeable difference between gravity conditions (7.7±2.4 µV in weightlessness versus 10.7±4.4 µV on Earth for P1 and 7.0±2.6 µV in weightlessness versus 15.5±6.8 µV on Earth for N1).

In order to test the influence of the stimulation frequencies, which were by nature different between the checkerboard and the virtual navigation paradigm, we examined the latency and amplitude of the P1 and N1 responses in the control experiments conducted on the ground, where both visual stimuli (checkerboard and 3D tunnel) were presented at 1.0 Hz. In this condition, the differences in the latency of the respective P1 and N1 components remained the same as those reported in the astronaut data. However, P1 amplitude for the checkerboard was higher for the 1 Hz presentation than those recorded at 3 Hz (10.0±4.7 µV at 1 Hz, versus 3.4±1.0 µV at 3 Hz; Chi^2^ = 4, df = 1, p<0.04). In contrast, P1 amplitude for the 3D tunnel remained in the same range (6±1.7 µV at 1 Hz versus 7.3±3.0 µV at the stimulus frequency in the virtual navigation procedure; Chi^2^ = 1, df = 1, p = 0.32). We may therefore conclude that the smaller amplitude of the checkerboard VEP compared to 3D tunnel VEP was due to a faster rate of stimulation.

In order to check whether the VEP reductions for the 3D tunnel in weightlessness was due to a difference in SNR we measured the reliability. We found no significant difference for the reliability for all the astronauts and trials for the checkerboard VEP (0.975±0.02 on Earth versus 0.974±0.03 in the ISS) and 3D tunnel VEP (0.976±0.002 on Earth versus 0.974±0.003 in the ISS) (F_(3, 36)_ = 1.44, p = 0.2477). These results suggest that the reported effects were due to physiological effects since the ERP signals recorded in both environments has the same noise characteristics.

### Theta-alpha rhythms related to the 3D tunnel changed in weightlessness

On Earth, the spectral analysis of single EEG trials recorded in astronauts revealed the presence of an event related synchronization (ERS) in the theta-alpha frequency band (3–13 Hz) occurring around the latency of P1 (∼100 ms) and extending up to the latency of the N1 peak, whatever the type of visual stimulus (Fig. 1*C,I*). Inter-trial coherence analysis (ITC) showed the presence of phase locking of the theta-alpha rhythm on the visual stimulation (Fig. 1*E,K*).

For the checkerboard, the ERSP and ITC values in the occipital loci (O2 channel) were conserved in weightlessness (ERSP_max_ of 1.2±0.6 dB on Earth before versus 1.5±0.6 dB in weightlessness; ITC_max_ of 0.41±0.14 on Earth before versus 0.45±0.11 in weightlessness) (Fig. 1*D,F*). Friedman ANOVA analysis showed no significant main effect of experimental conditions (before, during and after flight) on either (Chi^2^ = 0.13; df = 2; *p* = 0.94) or ITC (Chi^2^ = 2; df = 2; *p* = 0.37).

On the other hand, the same Friedman ANOVA applied to the data for the 3D tunnel showed significant effects of gravity conditions on both ERSP (Chi^2^ = 13.0; df = 2; *p*<0.001) and ITC (Chi^2^ = 15.86; df = 2; *p*<0.0004) indicating that both quantities were altered in weightlessness (ERSP_max_ of 5.54±2.77 dB on Earth before versus 2.5±0.7 dB in weightlessness; ITC_max_ of 0.76±0.18 on Earth before versus 0.45±0.19 in weightlessness) (Fig. 1*I,J*). Again, ERSP and ITC values returned to preflight values after arrival back on Earth (*p* = 0.09 for ERSP and *p* = 0.05 for ITC, Wilcoxon test). The analysis of the cortical activity in the frontal areas (i.e. as measured by the F8 electrode) showed that as for the occipital loci, ERSP and ITC corresponding to the checkerboard stimulation were conserved in weightlessness (Fig. 2*C,D,E,F*): ERSP_max_ = 0.94±0.56 on Earth versus 1.24±0.76 in weightlessness (Chi^2^ = 0.13; df = 2; *p* = 0.94); ITC_max_ = 0.34±0.08 on Earth versus 0.41±0.09 in weightlessness (Chi^2^ = 0.2; df = 2; *p* = 0.37). In contrast, a strong reduction in the phase-locking intensity for the 3D tunnel was observed (Fig. *2L,K*): ITC_max_ of 0.7±0.18 in weightlessness versus 0.9±0.07 on Earth (Chi^2^ = 8.98; df = 2; *p* = 0.01). However, in spite of these effects, the ERSP did not change in the frontal area (Fig. 2*J,I*): ERSP_max_ of 5.44±1.37 dB in weightlessness versus 7.18±1.85 dB on Earth (Chi^2^ = 1.64; df = 2; *p* = 0.44). It is thus the reduction of the phase locking of this oscillation that may explain the strong reduction of the ERP components when the 3D tunnel was presented in weightlessness (Fig. 2*G,H*).

**Figure 3:**
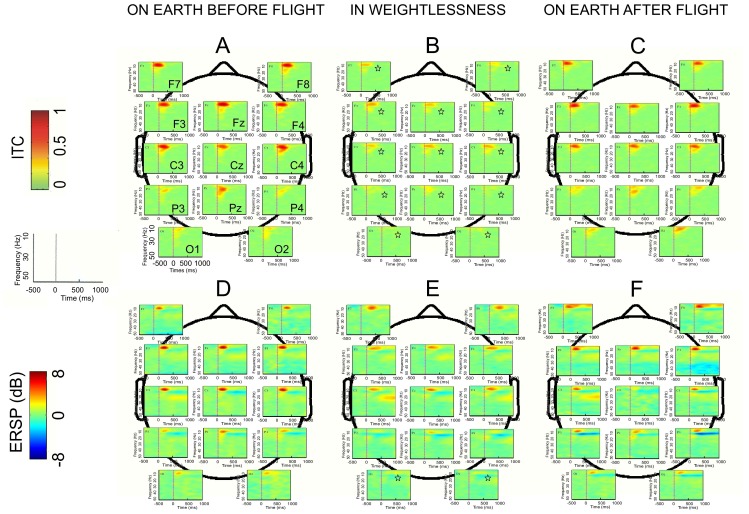
Effect of microgravity on the topographical representation of ITC and ERSP. ITC are represented in the upper part (***A*** *–* ***C***) and the ERSP in lower part (***D*** *–* ***F***). Grand average (n = 5) triggered by the 3D-tunnel presentation on Earth before flight (***A*** *,* ***D***) in weightlessness (***B*** *,* ***E***) and on Earth after flight (***C*** *,* ***F***). Each map corresponds to a single recording channel (from F7–F8 to O1–O2) disposed on the scalp. Statistical significance (Friedman ANOVA) p<0.05 is indicated by an asterisk.

Topographical analysis showed that the major reduction of the theta-alpha phase-locking was not restricted to frontal areas, being apparent throughout the entire scalp (Fig. 3*A,B,C*). On the other hand, the increase of theta-alpha power was conserved only in the frontal areas and progressively diminished from frontal to occipital positions in weightlessness (Fig. 3*D,E,F*).

**Figure 4:**
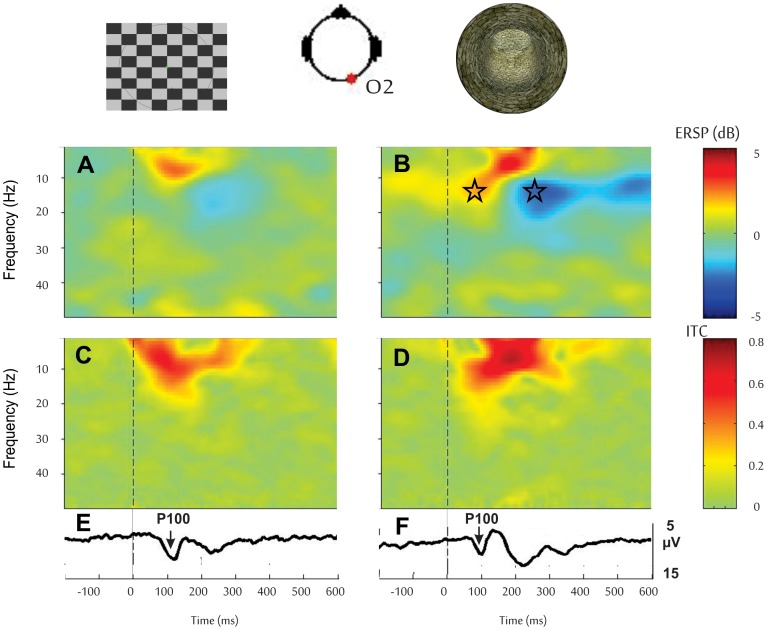
Comparison between checkerboard and 3D tunnel stimuli given at 1 Hz in control participants on Earth. From top to bottom, the ERS, ITC and ERP triggered by the checkerboard (***A*** *,* ***C*** *,* ***E***) and by the 3D-tunnel pattern (***B*** *,* ***D*** *,* ***F***). The triggers (vertical dashed lines) were given at time zero. The stars indicate stronger ERS in the upper alpha band (∼15 Hz) followed by a stronger ERD at about 200 ms in the upper alpha band (∼15 Hz) with respect to the checkerboard pattern.

As we did for the analysis of the ERP, we conducted ERSP analysis on occipital loci for trials where the checkerboard and the 3D tunnel stimulus were given at the same 1 Hz frequency rate in a group of control subject on the ground (Fig. 4). At the latency of P1 this ERSP analysis showed that the 3D tunnel evoked a stronger ERS in the upper alpha band (∼15 Hz) with respect to the checkerboard pattern (Fig. 4*C,D*): 2.3±0.9 dB versus 0.6±0.5 dB (Chi^2^ = 4.00; df = 1; *p*<0.04). There was no difference in the ITC response to either stimulus. It is interesting to note that although subjects were not asked to produce any behavioural response during this comparative testing, the ERSP map showed the presence of a significant ERD at about 200 ms in the upper alpha band (∼15 Hz, Fig. 4*B*), indicating a stronger neuronal excitation when the 3D tunnel was presented as compared to the checkerboard pattern (Fig. 4*A*): −3.5±1.3 dB for the 3D tunnel versus −1.4±0.2 dB for the checkerboard (Chi^2^ = 4; df = 1; *p*<0.04).

### The imaginary part of the frontal/occipital coherency changed in weightlessness

**Figure 5:**
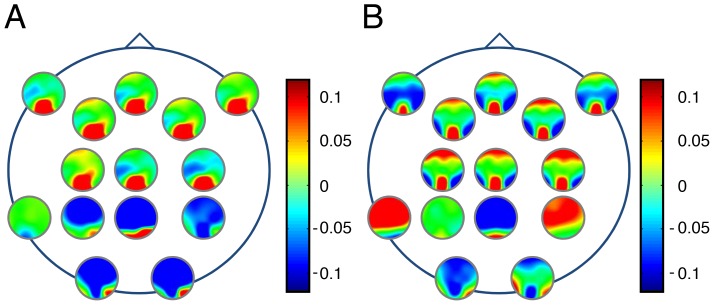
Effect of microgravity on the imaginary part of the coherency for the 3D-tunnel presentation. Non-parametric Statistical *t*-test on imaginary part of the 10 Hz coherency (n = 5) at the P1 latency (∼100 ms) evoked by the 3D-tunnel-image, on Earth (***A***) and in weightlessness (***B***).

**Figure 6:**
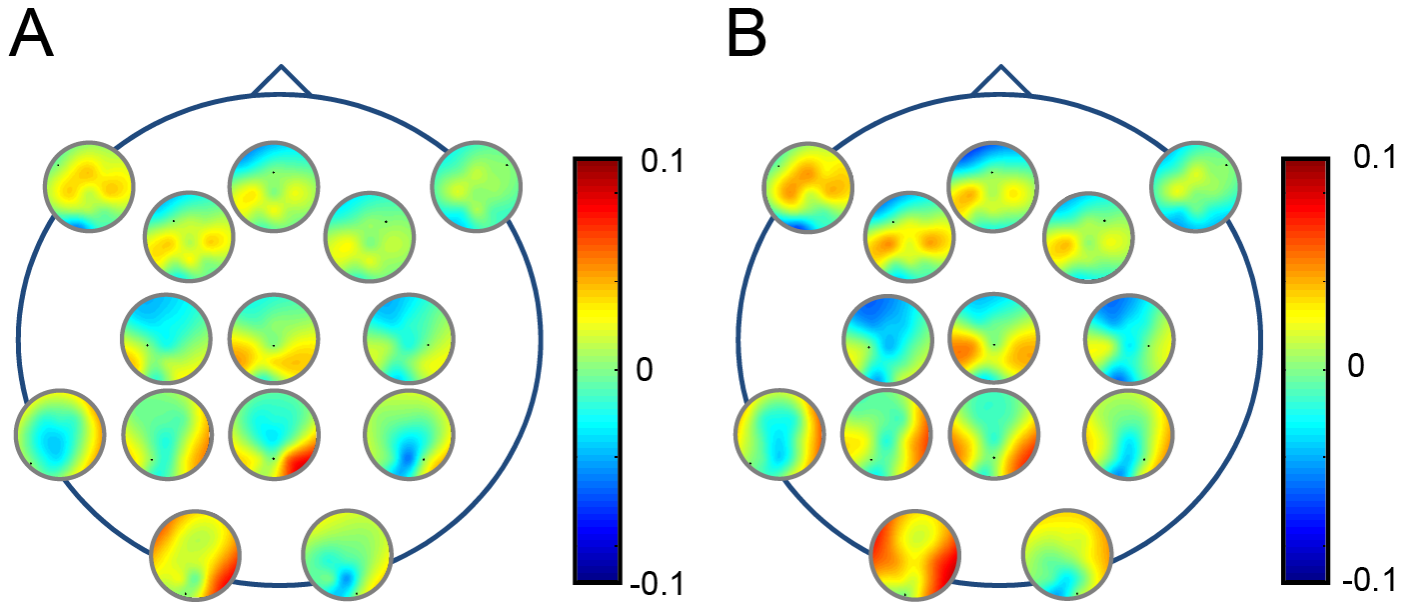
Effect of microgravity on the imaginary part of the coherency for the checkerboard stimulation. Non-parametric Statistical t-test on imaginary part of the 10 Hz coherency (n = 5) at the P1 latency (∼100 ms) evoked by the checkerboard stimulation, on Earth (***A***) and in weightlessness (***B***).

In order to better study the dynamical interaction, we analyzed the imaginary part of the coherency between the different cortical areas implicated in perception of the 3D tunnel presentation on Earth and in weightlessness. This analysis is summarized in Figure 5, illustrating the non-parametric statistic of the imaginary part of coherency calculated for the 10 Hz band between the fronto-central electrodes (Fz, F2, F3, F7, F8, Cz, C2, C3) at the latency of P1 (∼100 ms). We showed that the occipital electrodes (red surfaces, positive value) interacted with the frontal ones when the recordings were made on the ground (Fig. 5*A*), but that this coherency was significantly altered (*p*<0.05) in weightlessness (Fig. 5*B*). In addition, on the ground, the imaginary part of coherency was negative (blue surfaces) between the occipital (O1, O2) and the fronto-central electrodes (Fig. 5*A*). Weightlessness also altered this latter interaction (*p*<0.05) (Fig. 5*B*). Moreover, on the ground, central areas interacted primarily with occipital areas, while in weightlessness central areas interacted with both occipital and frontal areas (Fig. 5). The same analysis was performed on the checkerboard data (Fig. 6), but another configuration with less significant area emerged. Namely, the interaction observed on Earth between the occipital and the frontal electrodes for the 3D-tunnel presentation (Fig. 5*A*) was not present for the checkerboard (Fig. 6*A*). In the latter, only the occipital and temporal regions showed significant interactions (red surfaces). Moreover, in contrast to the 3D-tunnel, the dynamical interaction revealed by the imaginary part of the coherency corresponding to the checkerboard remained the same in weightlessness (Fig. 6*B*) reinforcing the preservation of this visual response in this condition.

### Fronto-occipital directionality was altered in weightlessness

**Figure 7:**
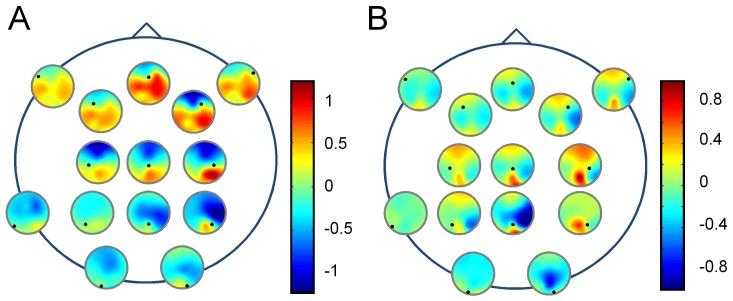
Effect of microgravity on directionality. Flow direction of information estimated by the phase-slope index on the imaginary part of the 10 Hz coherency for all pairs of channels averaged over all astronauts (n = 5) at the P1 latency (∼100 ms) evoked by the 3D-tunnel-image. The *i*th small circle is located at the *i*th electrode position and is a contour plot of the *i*th row of the matrix with elements ψ *ij*. On Earth (***A***), frontal areas are drivers and occipital areas are receivers. In weightlessness (***B***) flow is altered, splitting from the central area (drivers) into the frontal and occipital areas (receivers).

To estimate the direction of flow of information, we used the imaginary part of the coherency to compute the phase slope index, as proposed by Nolte et al [17]. Figure 7 shows the directionality index for the same data that was presented for the coherency analysis (Fig. 5). This confirms the existence on Earth (Fig. 7*A*) of an anterior-posterior flow of information toward occipital areas (receivers), whether the drivers were frontal or central (p<0.05). In contrast, in weightlessness (Fig. 7*B*) this directionality was altered and split in two divergent flows, from the central areas toward both the frontal and the occipital areas (p<0.05). We computed also the directionality for the checkerboard data, but no significant phase slope delays were found either on Earth or in weightlessness.

## Discussion

In summary, basic VEP responses induced by a checkerboard-reversal pattern (neutral stimulus) and the related theta-alpha phase locking were preserved in weightlessness. VEPs triggered by the presentation of a virtual 3D tunnel, and sustained by a theta-alpha phase locking and a fronto-occipital directional flux (top-down) on Earth, were, however, dramatically perturbed in weightlessness. It must be borne in mind that precise anatomical interpretation remains limited by the number of recording electrodes that preclude the use of an inverse model. To some extent, localization of the neural generators of ERSP and ITC from the scalp responses is somewhat speculative.

Non-specific factors, such as noisy environment in the ISS, stress, muscle artifacts and basic physiological factors (brain and body blood circulation difference), seem unlikely to be the source of the modifications to responses to the 3D image, given the preservation of the classical checkerboard VEP and the maintained level of psychophysical performance in the navigation task [27]. The phase-locking contribution to the VEP [28] induced by the presentation of 3D-tunnel on Earth was suppressed in microgravity, while those triggered by the checkerboard remained the same, suggesting the involvement of graviception in this process.

The major difference between the checkerboard and the 3D-tunnel tests is that in the latter situation the subject cognitively processed the visual information in anticipation of a 3D navigation task [5]. As the presentation of the 3D tunnel was followed by a navigation task that involves working memory, spatial orientation and eye-hand motor function, this visual stimulus may recruit the major pathways of the dorsal stream [29], namely, the parieto-medial temporal pathway including the major part of the parahippocampal and hippocampal formation which are focused on whole body motion in visuospatial frame of navigation [30], [31], [32]. In addition, the parieto-prefrontal and parieto-premotor pathways are respectively implicated in the top-down control of eye movements [33], [34] and in visually guided action [35]. There is a high probability, therefore, that the sustained activity in the prefrontal cortex [34], initiated here by the appearance of the 3D tunnel and playing the role of a driver in the directional flow of EEG signals, is implicated in the navigation task. The neutral checkerboard stimulus, on the other hand, would not recruit such pathways. This is also compatible with the new view that reconciles top-down and bottom-up effects on attention where salience, current goals and behavioral history are integrated in a functional map [36].

It is therefore quite logical that one might see differences in neural responses between the tunnel and checkerboard stimuli in weightlessness. Navigational processes would normally be carried out in a terrestrial gravitational frame of reference, which would implicitly take part in the evoked response. The unusual conditions of weightlessness appear to alter the normal workings of the underlying neural circuitry.

Specifically, the analysis of the phase-slope index of the imaginary part of the coherency presented here demonstrates the existence of a directional flow of information from frontal to occipital areas that could participate in a top-down action on the visual areas involving working memory [9]. The repeated exposure to the 3D tunnel followed by the navigational task and the related activation of working memory can influence the visual responses, as recently demonstrated in a target detection paradigm that showed a significant influence of long-term memory [37], [38], [39]. Interestingly, the direction of information flow that supports a frontal to occipital top-down action was altered and replaced by two directional flows from central areas toward frontal and occipital areas in weightlessness, providing an electrophysiological demonstration of a specific relocation of the driver along the dorsal pathway [29]. This reflects functional reorganization of frontal-central-occipital relationships to accommodate the absence of actual graviception by repositioning the oscillatory neural drivers and receivers.

Some differences in the configuration of the evoked responses argue in favour of the existence of specific neuronal populations activated by highly complex visual stimuli [40] or of perceptual grouping of V1 neurons supported by an increase in the rate covariation of neurons responding to features of the same object [41] depending on visual attention [42] or top-down modulation [43], [9], [44], [45]. Indeed, it has been demonstrated in the macaque that recognizable high-order stimuli induce larger activations in anterior visual and frontal areas while less meaningful stimuli induce greater activations in posterior visual areas [46]. The critical role played by contextual cues in object-specific responses can be applied to our virtual navigation task. In this environment, the gravitational frame of reference may implicitly participate in the visual perception and sensation of self-motion as an integral element of the general context [3], [5], [4]. Within this navigation network, visual signals are not only transmitted from lower-order areas (V1) to higher-order areas; re-entrant feedback or top-down influences are critically involved in early-evoked responses [13], [42], [43], [44], [47], [48], [49], [45]. The suppression of feedback or top-down mechanisms acting on the primary visual cortex [13], [50], [51] might therefore explain the effect of weightlessness on the 3D-tunnel-evoked responses. Interconnections between different networks related to visuospatial working memory and vestibular input such as the cingulate cortex may contribute to top-down modulation [52], [53]. Under this hypothesis, the top-down gravitational context would contribute to the channeling of visual information among the different possible neuronal populations, as recently demonstrated in prefrontal top-down modulation of early visual processing and working memory [13].

The present results suggest that the terrestrial graviception would implicitly take part in the physiological networking interaction characterized by the phase-slope index analysis of the imaginary part of coherency between the frontal and occipital cortex, while weightlessness may produce a basic interference in the network dynamics. As the coherency between two EEG-channels characterizes the linear relationship of the two time series at a specific frequency, it essentially measures how the phases are coupled to each other. By using the imaginary part of this measure we avoid false positive results due to the problem of volume conduction [16].

Although the limited number of electrodes precludes in the present case the determination by inverse modeling of the neural generators implicated in the fronto-occipital relationships, multiple equivalent dipole models were identified by Gramann et al. [54] during a similar task that also included the appearance of a virtual 3D-tunnel followed by a navigation task, on the basis of the event related spectral dynamics. Their study demonstrated the existence of occipital, parietal, precentral and frontal clusters of neural generators that explained the recorded ERS and ERD in the theta, alpha-mu and beta rhythms that were already active just after the presentation of the virtual tunnel [54]. These data reinforce the results presented here about the existence of a phase delay between occipital and frontal 10 Hz oscillations revealed by the coherency and directionality analysis and corroborate a top-down modulation of the occipital cortex by the frontal one.

We may therefore propose that the fronto-occipital interaction observed on the ground represents a mechanism of binding in the global networking involved in this active perception. The existence of a selective spatiotemporal coupling between dynamic motor representations and neural structures involved in visual processing was recently demonstrated [12]. This process could also be present in virtual navigation task. In this environment, the gravitational frame of reference may implicitly participate in the visual perception and sensation of navigation by the activation of frequency specific oscillation subtending interaction between the frontal and occipital network. It was proposed that different cortical areas combine signals with different modalities into a common spatial frame [55], [56]. Depending on the functional context these multiple sensory inputs are dynamically re-weighted to maintain behavioral goals [55], [12]. Phase coupling between different cortical and subcortical oscillations may provide the physiological foundation for keeping the spatial frame into a stable state. The present results could be integrated in the concept of synchronized resonances. As described for 40 Hz oscillations in the auditory domain [57] the phase coupling between the 10 Hz of the fronto-central and occipital areas may be viewed as a more global mechanism, working in parallel to the processing of the stimuli along the visual pathway. The phase-locking of this rhythm allows the placement of the 3D tunnel image in the temporal and environmental context, taking into account the intrinsic functional state of the brain at the arrival time of the stimulus. Therefore, the specific effect of microgravity on the 3D tunnel-evoked responses may be explained by the suppression of a top-down mechanism supported by the 10 Hz oscillatory interaction dependent of natural gravity and acting on the primary visual cortex.

An eventual role of general attention deficit related to microgravity can be ruled out in explaining our results as we did not find any significant differences in the error rates and response times related to the virtual navigation task [18]. This reinforces the idea that the specific alteration of the 3D-tunnel VEP was due to a direct gating effect on visual cortical areas provided by the absence of graviceptive and vestibular afferents in weightlessness. Such gating has been found in patients with vestibulopathy where cortical visual motion processing was suppressed [51].

The ERSP and ITC topographical analysis demonstrated a functional link between the alteration of the theta-alpha phase-locking process throughout the entire scalp and a disturbance of the top-down processing. Conservation of the early power increase in the same frequency band in the frontal region (but not in the occipital region) enhances the specificity of the alteration and excludes a decrease in awareness during tunnel presentation. As the role of alpha oscillation in visual evoked responses is well established [28], [58], [44], the fact that the alpha power during the eye-closed state increased in weightlessness and that both the gain of the ERD and ERS during the arrest reaction increased when the eyes were closed [59] rules out the existence of a general weakness in alpha rhythm generation in weightlessness.

In conclusion, the present study shows that in weightlessness, although the classical checkerboard VEP were preserved, responses evoked by the image of a 3D tunnel image presented at the start of a virtual navigation task were significantly altered. This alteration consisted of a rhythmic perturbation accompanied by a marked reduction in the phase locking of theta-alpha oscillations and a reorganization of the fronto-occipital directional flow of the 10 Hz oscillation that is present on Earth. Such effects demonstrate that a top-down modulation is exerted by gravity-related sensory inputs on visual inputs involved in tasks of virtual 3D navigation.
